# Implementations of translational medicine

**DOI:** 10.1186/1479-5876-3-33

**Published:** 2005-08-30

**Authors:** Kai-Christian Sonntag

**Affiliations:** 1 Harvard Medical School, Center for Neuroregeneration Research, MRC 102, McLean Hospital, 115 Mill Street, Belmont, MA 02478, USA

## Abstract

New developments in science are rapidly influencing and shaping basic and clinical research and medicine. This has led to the emergence of multiple opportunities and challenges on many levels in the bio-medical and other associated fields. To face these opportunities and challenges, new concepts and strategies are needed. These can be provided by translational research/medicine as an integrative concept based on a multidirectional understanding of research and medicine embedded in a socio-economical environment. Although the implementation of translational research/medicine faces many obstacles, some of its goals have already been part of new programs in local institutions and in medical or scientific societies. These implementations are important in creating a unified national and international system of translational research/medicine.

The rapid evolutions in science have generated a tremendous spectrum of new technologies and tools in both basic and clinical research/medicine. This includes the constant improvement of old and the discovery of new diagnostics and therapies, which increasingly contain and integrate elements from different fields, such as biomedical and other sciences, modern and traditional medicine and various technology branches. In addition, the application of these developments in clinical settings have created a "feed-back-loop" providing crucial information about their feasibility and success in improving human health. This network of scientific and clinical research/medicine has become one of the factors in shaping modern societies not only by being a major economical factor (see [1] for details), but also by challenging basic values and traditional thinking. To face the emerging challenges of creating a balanced and effective healthcare system, new concepts are needed for providing a framework of integrative strategies and solutions that efficiently combine basic and clinical research/medicine.

So far, translational research/medicine has rather been a linear concept rooted in traditional (academic) approaches to provide therapies for diseases (from bench to bedside), while paying little attention to patient-oriented research that involves understanding the underlying cause of disease and its treatments (from bedside to bench). Moreover, not much attention has been paid to many socio-economical aspects that are associated with research or medicine. Therefore, new definitions based on a bi- or multi-directional understanding of translational research/medicine have been proposed [2-5] and, recently, a strategic outline to successfully implement the goals of this new concept has been outlined in an article by Hörig et al. in Nature Medicine [1].

This commentary aims to add a few additional aspects by emphasizing two major factors that strongly influence and, in turn, are influenced by translational research/medicine: (1) The rapid evolution of new technologies and therapeutic approaches, and (2) examples of attempts to create programs, which are based on a translational understanding of basic and clinical research and/or medicine.

In the past two decades, biomedical and other research fields like gene therapy, the human genome project (HUGO), stem cell research, cloning, nanotechnology and others have revolutionized medicine and generated entirely new fields and approaches in treatment of diseases [6]. Thus, it is becoming more and more obvious that new forms of therapies, such as gene- or cell-replacement-therapy, will have a place in future therapeutic approaches. In addition, the rapid expansion of bio- and other technology development are expected to lead to an advanced understanding of diseases [7,8]. These new fields not only offer multiple new opportunities in the research and biomedical sector, but also require attention on multiple other levels involving religious and ethical issues [9] including new definitions of life, disease, and treatment, the power and limitations of technology, the availability of resources, and the adjustment of social and political actions.

How can these fields be combined and integrated in the concept of translational research/medicine? Are there any attempts to develop programs based on a "translational" thinking in research and medicine? So far, the concepts of translational research/medicine have been described and discussed in the context of different medical fields, e.g. in obstetrics and gynecology [10], radiation therapy and radiobiology [11,12], cancer therapy [8], psychiatry [13], pain research [14], gastroenterology [15] or in the broader context of research and medicine [16]. And, so far, it seems that there is an overall agreement in defining the goals of translational research/medicine and delineating the obstacles in their implementations [1,17-19]. In addition, many of these articles have provided suggestions and strategies how to translate these concepts into practice (e.g. [1,8,12,20-23]). However, it should be emphasized that translational research/medicine does not affect specialized fields alone. Rather it should be an integrative concept that, with implications to everybody in the field, should be part of a broader understanding of how research and medicine should be organized. A major part of these processes refers to education and training opportunities and there have been examples published how this can be implemented by personal experience (e.g. [16,24]), by the quest for specialized institutions (e.g. [20]), or by using existing educational systems (e.g. [25-29]). It should also be noted that scientific/medical societies have started to adopt and integrate many aspects of the concept of translational research/medicine in their agendas and are in the process to find new creative and innovative strategies on multiple levels, including education, public discussions, funding opportunities, career development, etc. This especially applies to societies, which integrate a vast spectrum of bioresearch, biotechnology and biomedical fields such as the American Society for Gene Therapy (ASGT; ) or the International Society for Stem Cell Research (ISSCR; ). On the local level, there are existing clinical programs that aim to improve the clinical education by emphasizing translational thinking (e.g. [25,27-30]) or other programs with a broader approach of being integrative between researchers, clinicians and health care providers. The latter programs – like the medical societies – offer new opportunities to facilitate collaborations between researchers and clinicians and represent a platform to process the multilevel challenges emerging from the rapid advances in the biomedical fields. For example, the Harvard Medical School has launched two new centers, the Harvard Center for Neuroregeneration (HCNR; ) and the Harvard Stem Cell Institute (HSCI; ), whose goals are to create an environment that combines investigators on multiple levels and address many features of translational research/medicine like identification of new and alternative funding opportunities including academia-industry synergies, healthcare delivery, the improvement of infrastructural hurdles by creating core facilities, training opportunities, etc. These "enterprises" require significant logistical efforts and often rely on established systems, which can provide, space, informatics, administration, and skilled personnel. Although these are local efforts and might not reflect the overall situation, it shows that efforts are made and many aspects of translational research/medicine can be realized. The experiences gained from these local "enterprises" might be then translatable and integrated into a bigger national and international network creating a more unified system of translational research/medicine.

In summary, translational research/medicine is not a single standing idea that can be applied whenever it is needed. Rather, it is a concept that needs the attention from everyone and should be the foundation of a modern understanding of health provision. It is essential to face the challenges emerging from the rapid advances in the biomedical and other associated fields. Its implementation needs a multilevel effort in many different areas, which together create a health system as a whole. Some of the goals in translational research/medicine are already part of new programs in local institutions and in medical or scientific societies. These implementations are important in creating an overall and unifying network of translational research/medicine.
